# Advances in Virus-Directed Therapeutics against Epstein-Barr Virus-Associated Malignancies

**DOI:** 10.1155/2012/509296

**Published:** 2012-03-05

**Authors:** Sajal K. Ghosh, Susan P. Perrine, Douglas V. Faller

**Affiliations:** Cancer Center, Boston University School of Medicine, Boston, MA 02118, USA

## Abstract

Epstein-Barr virus (EBV) is the causal agent in the etiology of Burkitt's lymphoma and nasopharyngeal carcinoma and is also associated with multiple human malignancies, including Hodgkin's and non-Hodgkin's lymphoma, and posttransplantation lymphoproliferative disease, as well as sporadic cancers of other tissues. A causal relationship of EBV to these latter malignancies remains controversial, although the episomic EBV genome in most of these cancers is clonal, suggesting infection very early in the development of the tumor and a possible role for EBV in the genesis of these diseases. Furthermore, the prognosis of these tumors is invariably poor when EBV is present, compared to their EBV-negative counterparts. The physical presence of EBV in these tumors represents a potential “tumor-specific” target for therapeutic approaches. While treatment options for other types of herpesvirus infections have evolved and improved over the last two decades, however, therapies directed at EBV have lagged. A major constraint to pharmacological intervention is the shift from lytic infection to a latent pattern of gene expression, which persists in those tumors associated with the virus. In this paper we provide a brief account of new virus-targeted therapeutic approaches against EBV-associated malignancies.

## 1. Introduction

Epstein-Barr virus (EBV) infection is ubiquitous in human populations worldwide. EBV infection in children and adolescents usually leads to a self-limiting lytic infection, designated as infectious mononucleosis (IM) [1, 2]. However, in immunocompromised individuals, such as those with X-linked lymphoproliferative disease (XLP) [3, 4], EBV infections often progress unchecked and are lethal. EBV is invariably associated with nasopharyngeal carcinoma (NPC) [5], African Burkitt's lymphoma (BL) [6], posttransplantation lymphoproliferative disease (PTLD) [7–10], and less often with a number of other human malignancies such as Hodgkin's lymphoma (HD) [11], and non-Hodgkin's lymphomas (NHL). In addition, EBV is found in a fraction of gastric carcinomas [12, 13] and carcinomas of the breast [14–16]. Although EBV has been identified in these latter tumors, it remains controversial whether EBV is causally-related to their development. Nonetheless, multiple studies have clearly demonstrated that the presence of EBV in these tumors confers a poorer prognosis [17–22]. 

In the mid-Eighties, the technique of random cleavage of the terminal repeat region of the EBV genome was employed as a method of identifying clonality of the virus episome population in infected cells or tissues [23, 24]. During EBV lytic replication (described later), long head-to-tail concatameric DNA is produced from the circular episomal DNA, which is then cleaved randomly within the terminal repeat region by viral-encoded terminase, leading to the production of virus particles with varying length of their terminal repeats [25]. Latently infected B-cells, however, do not produce virus particles and the circularized EBV episomal genome replicates in coordination with the division of cellular genome, producing exact copies of the viral genome in daughter cells. Multiple studies using this type of analysis clearly demonstrated that the EBV genome in many of the EBV-associated tumors, such as BL [26], NPC [23], and HD [11], is clonal in nature, strongly suggesting that these tumors developed from a single progenitor cell that was already infected with EBV, lending further support to the concept that EBV may be causally related to the genesis of many of these tumors.

## 2. EBV Infection and Replication

EBV transmission usually takes place through the mucosal secretions of the mouth of an infected individual. Primary infection of epithelial cells of the oropharynx leads to active production of virus particles with shedding of the virus in saliva. Although the EBV-epithelial cell attachment process is not fully understood, the neighboring B-cells subsequently become infected via interaction of the EBV surface protein gp350 with the lymphocyte receptor CD21, however, such infections are often nonproductive. Active or “lytic” replication of EBV induces lysis of infected cells concurrent with production of virus particles, whereas latent replication of EBV does not. EBV is a member of the gamma herpesvirus family, with a large 172 Kb double-stranded linear DNA genome encoding nearly 100 genes. Most of these genes are expressed during lytic-phase replication, whereas only a maximum of eleven viral genes are expressed during latent-phase replication. The up to eleven EBV gene products that are expressed in latently infected cells (the number depends upon the type of latency) include six nuclear antigens (EBNA1, EBNA2, EBNA3A-3B-3C, EBNA-LP), three latent membrane proteins (LMP1, LMP2A-2B), the BARF0 protein, generated from BART transcripts, and two small noncoding non-poly-A RNAs (EBER1 and EBER2). Primary EBV infection results in strong humoral and cellular immune responses. IgM antibodies against EBV surface protein (gp350) are easily detectable in the serum during primary infection, which is then eclipsed by a steady state level of IgG antibody over the ensuing months and beyond [27]. 

The symptoms of acute infection, such as IM, subside within few weeks as EBV enters a latent replication mode. EBV subsequently generates a life-long persistent infection in all infected individuals. Seroepidemiological surveys demonstrate that more than 90% of humans are positive for EBV antibody [28, 29]. The majority of infected individuals bear cytotoxic T cells directed against the virus, and at any given time only a small proportion of resting B cells are latently infected with the virus (one or two in a million) [30, 31]. EBV infection of primary human B lymphocytes *in vitro* leads to their immortalization and the development of continuously growing lymphoblastoid cell lines (LCL) [32]. In a healthy individual, however, a strict equilibrium between proliferation of EBV-infected B cells and immune surveillance is maintained [33]. In the setting of immunodeficiencies (whether hereditary or induced), however, this equilibrium is lost and the resulting unchecked proliferation of virus-immortalized B cells can then lead to the development of lymphoma, such as XLP or PTLD.

## 3. Therapies against EBV Diseases

Existing therapeutic approaches for EBV-associated diseases are broadly categorized into three groups, as shown in Table 1.

### 3.1. Pharmacological Therapy

**Figure 1:**
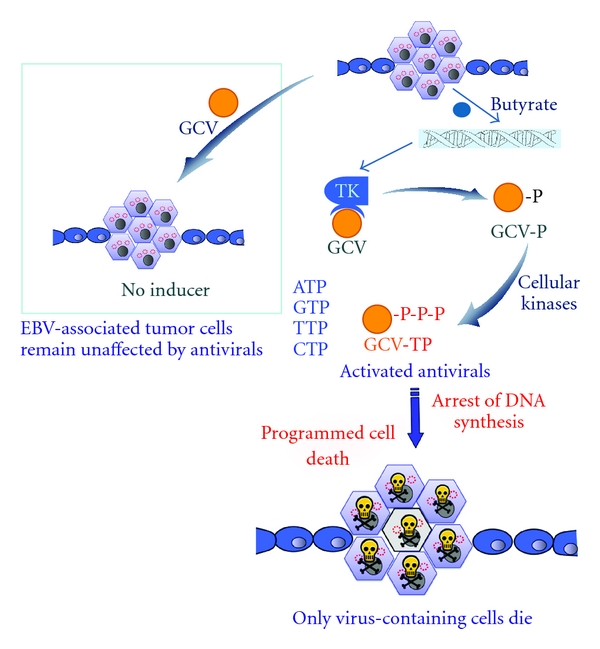
Schematic diagram of combination therapy approaches in EBV malignancies. EBV maintains latent replication in tumor cells and these tumor cells are not susceptible to anti-herpesvirus prodrugs, such as GCV. In the presence of lytic-phase gene expression-inducing agents such as butyrate, the latent EBV expresses thymidine kinase (TK) which converts the prodrug GCV to GCV-P, which is then converted to the (cytotoxic) triphosphate form by cellular kinases. During DNA replication, the triphosphate form of GCV is then incorporated into genomic and viral DNA, causing chain termination, cell-cycle arrest, and apoptosis of the EBV-infected cells.

Nucleoside-analog anti-herpesvirus drugs, such as ganciclovir, acyclovir, or famcyclovir, are moderately efficient in suppressing virus replication and virus shedding during diseases characterized by acute or lytic replication of EBV. They are not active in EBV-associated malignancies, however, because in EBV-associated malignant diseases the EBV maintains a latent state of replication. These commonly-used antiviral agents are prodrugs, and require conversion to their active form by virus-encoded kinases before they can be effective (see Figure 1). For the Epstein-Barr virus, however, these viral kinases are expressed only during lytic replication. 

### 3.2. Immunotherapy

Immunotherapeutic approaches have been studied in clinical trials for a number of years, with success in some cases. Normally, the host's CD4^+^ and CD8^+^ cytotoxic T cells and natural killer (NK) cells play an important role in killing EBV-infected cells during primary infection. Although EBV can often be cultured from throat washings of previously infected individual, continuing CTL immunosurveillance in normal individuals is quite efficient in controlling subsequent reactivation of EBV infection. Vaccination with recombinant gp350 viral glycoprotein or CTL epitope-based peptide has been successful in generating viral immunity in animal models [34–36] and may in the future prove useful in areas that are endemic for EBV malignancies (such as China and southeast Asia). Clinical vaccine trials in healthy individuals demonstrated the appearance of neutralizing anti-EBV antibodies in vaccinated individuals [37, 38]. However, the ubiquitous nature of EBV infection but low incidence of malignancies arising from the infected individuals makes prevention of EBV-associated malignancies of lesser importance than the control of the malignancy once it has occurred. Adoptive transfer of EBV-specific CTLs from an EBV-positive donor to the transplant recipient has been utilized in a limited fashion in the treatment of PTLDs and other solid tumors [39–42]. CTLs may also be isolated from a recipient's own lymphocytes, expanded *in vitro*, and infused back into the patient [43, 44]. These approaches have provided some clinical benefit in certain highly selected patients, particularly in the treatment of PTLD [40, 45, 46]. However, adoptive transfer of EBV-specific CTLs has not been as effective in patients with NPC or HD [47]. This approach is constrained by the availability of donor lymphocytes, and the long time required for the *in vitro* processing and expansion of the CTL. Furthermore, the requirement for prior lymphodepletion for *in vivo* CTL expansion is also a major obstacle. Radiation and chemotherapy-induced lymphodepletion often lead to multiple unwanted side effects. Recently, CD45 monoclonal antibodies are being used to induce a short-term lymphodepleted environment without unwanted side effects, allowing subsequent expansion of infused EBV-specific CTLs [48]. A recent comprehensive review of EBV-specific T-cell therapies currently under investigation is available [49]. 

## 4. Virus-Targeted Therapies

In most EBV-associated malignancies, all or nearly all of the tumor cells contain the viral genome. Furthermore, at any given time, the number of EBV-infected nontumor cells present in other physiological compartments of the host is usually very low, and for B cells is on the order of one in a million. This provides a unique opportunity to develop therapeutic strategies utilizing the presence of the viral genome of EBV in the tumors as an essentially “tumor-specific” target. One of the virus-targeted therapeutic strategies is based on the concept that EBV-containing cells will die if lytic replication can be induced. Other strategies employ selective expression of toxins in EBV-infected cells or preventing the function of EBV latent gene products that are linked to oncogenesis (Table 2). Elimination of episomal EBV genomes by low dose hydroxyurea treatment has been shown to decrease the tumorigenic potential of Akata cells of BL origin, both *in vitro* and in SCID mice [50]. When two patients with AIDS-related (EBV-positive) primary lymphoma of the central nervous system were treated with low dose hydroxyurea, their median survival compared to historical controls increased by almost 18 months [51]. The effectiveness of this approach in a controlled clinical trial, however, has yet to be evaluated. Expression of antisense RNA against the EBV LMP-1 protein has been shown to reduce LMP-1 expression in LCLs and concomitantly inhibit cell proliferation and stimulate apoptosis [52]. As EBNA1 is a viral transactivator expressed in all latently EBV-infected tumor cells and utilizes the OriP promotor for its activity, several studies have utilized an OriP-based vector to direct the expression of cellular toxins, such as driving cytosine deaminase expression (which converts the prodrug 5-flurocytosine to cytotoxic 5-flurouracil), or the herpes simplex virus TK, to make the cells susceptible to nucleoside analog antiviral drugs [53, 54]. Targeted delivery of these EBV-dependent vectors specifically to the tumors cells, however, remains a serious and unresolved challenge.

The most significant advancement in virus-targeted therapies for EBV malignancies is undoubtedly the combination therapy that is based on artificial induction of EBV lytic-phase gene expression, followed by exposure of the tumor cells to anti-herpesvirus drugs. It was established thirty years ago that induced expression of the herpes simplex virus TK enzyme renders cells susceptible to prodrugs such as ganciclovir or acyclovir [55]. Gutiérrez et al. first utilized this approach in EBV lymphoma cells by transfecting a Zta expression plasmid under the control of an OriP promoter. This plasmid also expressed HSV-TK. Transfection of this plasmid in the EBV-positive BL cell line P3HR1 induced lytic-phase gene expression, and in the presence of ACV, significantly reduced the growth of P3HR1 cells [56]. In two other studies, adenoviral vectors containing a BZLF1 or BRLF1 expression cassette under the control of the CMV promoter were used to infect the EBV-positive BL cell line, an NPC cell line, and an EBV-transformed gastric carcinoma cell lines [57, 58]. The BZLF1 and BRLF1 proteins both initiate the lytic replication cycle of EBV. In both of these studies, adenoviral vector-mediated BZLF1 and BRLF1 expression induced EBV-lytic phase proteins, such as BMRF1, in all the cell lines tested, whereas the empty adenovirus vector (expressing only GFP) had no such effect. When these adenovirus vectors were injected directly into xenografted tumors in SCID mice induced by the BL or NPC lines, BZLF1 and BLRF1 expression in the tumors was readily detected. There was a substantial coincident reduction of xenograft tumor size in the BZLF1- or BRLF1-adenovirus vector-injected tumors compared to controls [58]. The addition of GCV, however, did not appear to have any additional effects on tumor burden. The authors speculated that high level expression of EBV immediate-early genes, such as BZLF1 or BRLF1, in normal cells could be toxic and acknowledged that there is no reliable gene delivery system yet available to target only EBV-containing cells [59]. 

The mechanisms underlying induction of EBV lytic replication out of latency using chemical inducers such as phorbol esters (PMA), 5-Azacytidine (5-Aza-C), sodium butyrate, and other agents have been studied for decades [60–64]. These specific agents are known to induce or support the expression of EBV immediate-early genes. In the case of PMA, this effect is the result of protein kinase C-mediated activation of the Jun-Fos proteins and their interaction with AP-1 binding sites on the regulatory elements of the EBV IE genes [65]. Exposure to 5-Aza-C removes the transcriptional block on expression of the EBV genome during latency, caused by extensive CpG methylation [66]. Butyrate facilitates reactivation of latent EBV by allowing remodeling of the chromatin-like structure of the EBV genome, through its histone deacetylase (HDAC) inhibitory activity [67, 68]. The recent discovery that butyrate and other HDAC inhibitors (HDACi) can also induce demethylation and reactivation of methylated, silenced genes through repression of DNA methyltransferase 1 DNMT1 [69] may also contribute to their activity in inducing EBV lytic-phase gene expression. Combination therapy using a chemical inducer of EBV lytic-phase gene expression (arginine butyrate) and the anti-herpesvirus prodrug GCV was first tested on lymphoma cells derived from the EBV-positive lymphoma from a lung transplantation patient [70]. Arginine butyrate exposure induced the lytic-phase gene and protein TK in these lymphoma cells, and, in combination with GCV, inhibited the proliferation of the tumor cells in a dose-dependent manner. This *in vitro* activity led to a protocol for treatment of the lung transplant patient (who was already receiving GCV with no effect on the lymphoma) with 750 mg/kg/day arginine butyrate therapy for 15 days. The combination therapy was well tolerated by the patient, as no additional toxicity was observed. Although the patient eventually succumbed to an unrelated Aspergillus infection, pathological examination revealed substantial necrosis of the tumor following the treatment [70]. This study demonstrated for the first time the feasibility of combination therapy in the treatment of EBV malignancies in human subjects. Shortly thereafter, other inducers of EBV lytic replication were investigated *in vitro* and also in xenografted EBV tumor models in mice. Westphal et al. employed *γ*-radiation or sodium butyrate treatment to induce EBV lytic-phase gene expression in LCLs, BL cells Akata and Jijoye, and in an EBV-transformed gastric carcinoma cell line AGS [71]. Although the extent of EBV reactivation was variable within different cell lines, on average, 400 cG irradiation induced the immediate-early BMRF1 protein in all of the lines. Induction of BMRF1 by sodium butyrate (up to 2.5 mM) was inconsistent in this study, although a combination of butyrate and irradiation markedly induced BMRF1, irrespective of the cell lines tested. When xenografted tumors in SCID mice induced by these EBV-positive cell lines were exposed to a single dose of *γ*-radiation (400 cG), or were injected intraperitoneally with 500 *μ*L of 50 mM sodium butyrate, the BMRF1 protein was induced in the tumors, although less efficiently by butyrate than by *γ*-irradiation. A subset of the xenografted animals that were exposed to *γ*-radiation were also treated with GCV and followed over a month. Tumors in three out of four mice in this group did not progress in size. This study therefore supported the earlier findings in the clinical trial that the combination of induction of EBV lytic-phase proteins and treatment with anti-herpesvirus drugs may be a useful therapeutic approach. 

Based on the results of our previous combination treatment approach for the EBV lymphoma in a lung transplant recipient [70], we initiated a phase I/II clinical trial with ten patients, all of whom had EBV-positive lymphoid tumors which were refractory to conventional chemotherapeutics or radiation therapy. The types of tumors in the patients enrolled in the trial included PTLD, B-cell NHL, cutaneous T-cell lymphomas, T/NK lymphomas, and HD. The trial design utilized an intrapatient dose escalation of arginine butyrate combined with a standard dose of GCV. Preliminary results of this study, published in 2001 [72], demonstrated that five out of 10 patients had complete clinical responses and two additional patients had partial responses. Complete necrosis of the EBV lymphoma was noted in two out of the three patients in which pathological analyses were carried out. Analysis of the patient-derived tumor cells in culture again confirmed induction of EBV TK expression and resulting susceptibility to GCV [73]. A complete report of this multicenter multinational trial, with five additional patients included, was reported more recently [74]. Altogether, 10 out of 15 patients, all of whom had tumors refractory to all conventional therapies, showed significant anti-tumor responses, with 4 complete responses and 6 partial responses. Although complications from rapid lysis of the tumors did occur in 3 patients, this study demonstrated that the combination of arginine butyrate and GCV was well tolerated in a broad spectrum of patients. The maximum tolerated dose of arginine butyrate was found to be 1250 mg/kg/day. This controlled phase I/II trial paved the way for studies of this combination therapy approach in larger cohorts. It is also noteworthy that response to this regimen occurred in patients with multiple diverse types of lymphomas, including both lymphomas in which EBV was clearly a causative agent (PTLD), and in tumors where the etiological role of the virus was less clear (B-cell NHL, cutaneous T-cell lymphomas, and T/NK lymphomas). It appears that the presence of the virus in latent form alone is sufficient to render the tumor cells sensitive to this therapeutic approach. *In vitro* studies, in which the virus is artificially introduced into the tumor cells, have confirmed this (Ghosh and Faller, unpublished).

Several other studies have investigated the potential of other lytic-phase gene expression-inducing agents in preclinical studies, using EBV+ lymphoma cell lines. Some of these studies also utilized mouse models to test the reproducibility of their cell culture findings. DNA methyl transferase inhibitors, HDACi, radiation therapy, proteasomal inhibitors, B-cell receptor-blocking antibodies, and chemotherapeutic drugs were evaluated in these reports. A list of these studies is presented in Table 3. The studies all demonstrated varying degrees of activity in the various model cell lines. The study of Fu et al. [84] included an interesting variation which utilized an ^125^I-labeled nucleoside analogue, 2′-fluoro-2′-deoxy-ß-D-5-iodouracil-arabinofuranoside (FIAU), to visualize antiviral agent uptake in EBV+ tumors, by specialized CT-imaging. In this study, EBV lytic-phase gene expression in xenografted EBV+ BL tumors in SCID mice was induced by the chemotherapeutic proteasomal inhibitor bortezomib. Biodistribution analyses indicated preferential accumulation of FIAU in the tumors of the bortezomib-treated animals by 48–72 hr, but not in the vehicle-treated animals. The experimental radiopharmaceutical ^131^I-FIAU was used in a later study by this group which demonstrated xenografted EBV-tumor regression in SCID mice in response to combination treatment with ^131^I-FIAU and bortezomib by CT imaging [81]. 

While the basic concept of combination therapy has been tested in multiple *in vitro* studies and animal studies, and proof-of principle has been documented in the clinical trials, the optimal pharmacological inducer of EBV lytic-phase gene expression has not yet been determined. Ideally, this inducer should be highly active in inducing EBV lytic-phase genes and should have excellent bioavailability. In our clinical trial, we demonstrated that butyrate acts as an effective inducer of EBV lytic replication, and, together with an anti-herpesvirus drug, inhibits tumor growth and induces tumor regression [74, 76]. Because of its very short half-life *in vivo*, however, butyrate required continuous infusion over several days for therapeutic synergy with antivirals. In a recent study, we demonstrated that multiple short 6–8 hr exposures of P3HR1 cells to butyrate also efficiently induced EBV TK, and in the presence of GCV inhibited tumor cell growth [78]. This observation was translated into a clinical protocol to treat a rituximab-refractory EBV-positive PTLD patient who had received a cord-blood stem cell transplant for Hodgkin's disease [85]. A 5-day infusion of arginine butyrate and 21 days of GCV/valganciclovir treatment resulted in complete resolution of 4 out of 6 lesions in this patient, and decrease in the size of two other lesions. It is also noteworthy that this patient was symptomatic from EBV, CMV, and HHV-6 viremia, with very high plasma viral loads, despite ongoing administration of GCV/valganciclovir. After 1 cycle of combination therapy, these viral levels decreased by several logs or fell to undetectable levels, and her symptoms resolved, suggesting the applicability of this combination approach to multiple types of viruses in addition to EBV. 

**Figure 2:**
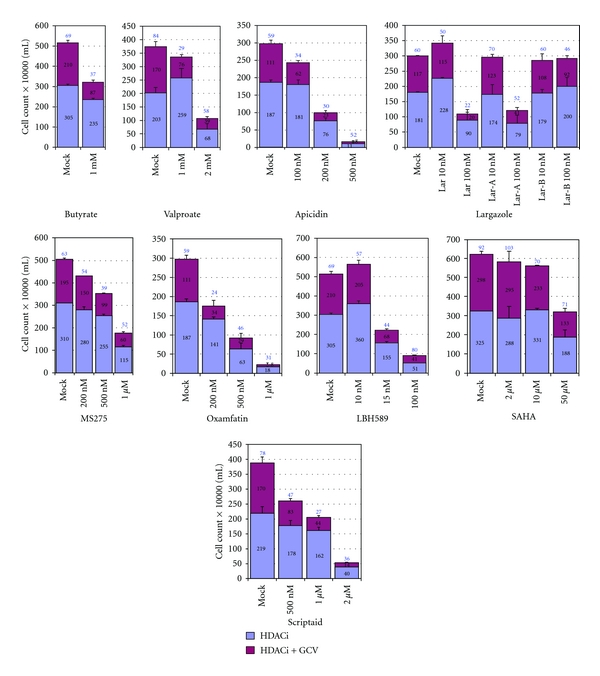
Cytotoxic activity of various HDACi in the presence of an anti-herpesvirus nucleoside analog prodrug, GCV. Three hundred thousand P3HR1 cells were exposed to either 40 *μ*M GCV or vehicle, and the indicated concentrations of individual HDACi, in a 1 mL volume in 24-well plates, in triplicate. Seventy-two hrs later, 800 *μ*L of the media was removed without disturbing the settled cells and 1 mL of fresh growth media containing GCV (40 *μ*M) was added and the cells, which were cultured for another 72 hrs. HDACi studied included butyrate, valproate, apicidin, largazole and its analogs, MS275, oxamflatin, LBH589, SAHA, and Scriptaid. The number above the HDAC+GCV bar represents the percentage of cells surviving, relative to the cultures exposed to that particular HDAC inhibitor alone (assigned a value of 100%). Error bars represent standard deviation.

**Figure 3:**
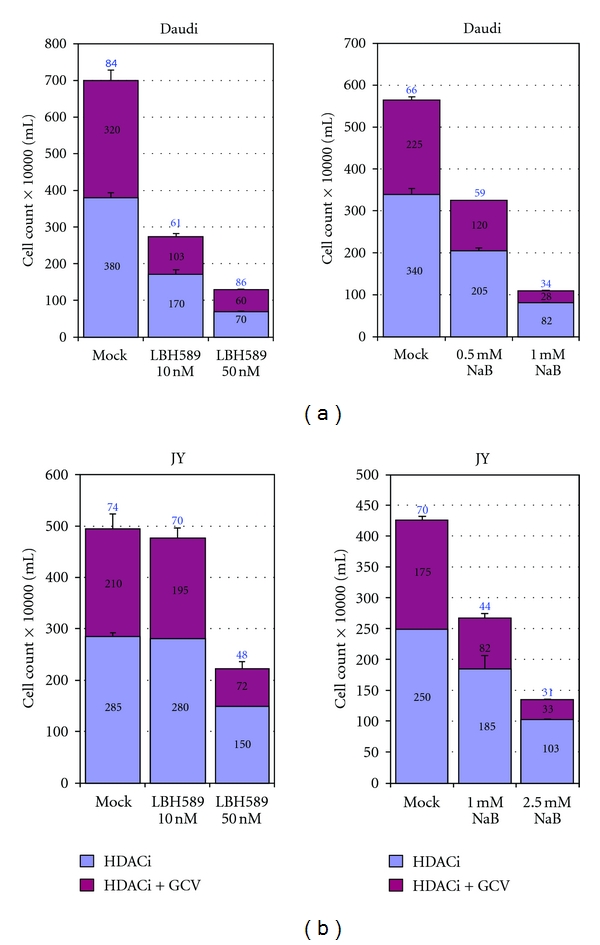
Effect of HDACi and GCV combination treatment on other EBV-positive lymphoma cells. (a) Effect of combination treatment on the BL line Daudi. Four hundred thousand cells per ml per well were cultured with 60 *μ*M GCV in the appropriate wells. Assay parameters were as described in the legend to Figure 2. (b) Effect of combination treatment on the EBV-transformed lymphoblastoid cell line JY. In this case, 200,000 cells per well were plated in the presence of 60 *μ*M GCV, or vehicle, as indicated. Experiments were repeated a minimum of three times and error bars represent standard deviation.

*In vitro* studies have demonstrated that HDACi are potent antiproliferative agents that cause cell-cycle arrest, apoptosis and/or differentiation of tumors [86, 87]. Furthermore, their preferential cytotoxic activity on tumor cells over normal cells suggested potential anticancer therapeutic application. In recent years, a number of HDACi have been tested in clinical trials. In addition, efforts have been made to develop more potent, or more HDAC class selective, HDACi. Although HDACi as a class are well-known inducers of EBV lytic-phase gene expression [88], only butyrate and valproic acid have been tested for their activity in treating EBV malignancies [70, 76, 77]. We have recently completed studies to test a variety of HDACi of different chemical classes, including some new and highly potent compounds, for their ability to sensitize EBV-lymphoma cells to anti-herpesvirus drugs. The HDACi studied included short-chain fatty acids (butyrate, valproate), hydroxamic acids (SAHA, oxamflatin, LBH589, scriptaid, PDX101), benzamide (MS275), a cyclic tetrapeptide (apicidin), and largazoles (originally isolated from marine cyanobacterium). With the exception of SAHA and PXD101, all of the other HDACi produced sensitization to GCV, and the combination caused cytotoxicity in EBV+ lymphoma cells (when used as a single agent, PDX101 itself exerted a strong cytotoxic effect on the cells [89, 90]). LBH589, MS275, and synthetic largazole derivatives were 10^4^ to 10^5^ times more potent in killing EBV+ lymphoma cells in presence of GCV, compared to sodium butyrate (Figure 2). The effective concentration of LBH589 was in the range of 50–100 nM, MS275 at 200–500 nM, and largazoles at 100–200 nM. The effectiveness of these HDAC-inhibitory (HDACi) compounds at such low concentrations makes them potentially applicable as sensitizers to antiviral therapeutics for the treatment of EBV-associated lymphomas. Furthermore, butyrate and LBH589 were also found to potently sensitize another BL cell line (Daudi) and a lymphoblastoid cell line (JY) (Figure 3). These findings therefore suggest that that these new and potent HDACi may provide alternative therapeutic options to butyrate, in combination with nucleoside antiviral agents, for the treatment of EBV-associated tumors.

## 5. Concluding Remarks

EBV-associated malignancies remain a significant health concern worldwide, with particularly higher incidences in southeast Asia and China. In Western countries, the incidence of EBV malignancies, and particularly PTLD, is also on the rise. The increasing use of solid organ or hematopoietic transplantation, especially in situations requiring intense immunosuppression, is most likely a major contributor to this increase in EBV-lymphomas. Furthermore, as referenced above, the presence of EBV in the common NHL and HD lymphomas confers a much poorer prognosis after conventional therapy, demonstrating the need for new therapeutic approaches.

The presence of EBV in these tumors represents a potential “tumor-specific” targeting opportunity for the development of new therapeutics. Among all potential therapeutic avenues explored to date for targeting the virus in EBV-positive tumors, pharmacological induction of EBV lytic infection coupled with an anti-herpesvirus agent appears to be most promising, as a variety of potential virus-inducing agents (some of which are logs more potent than butyrate) are now available, as are active and safe antiviral prodrugs. Furthermore, because a few of the new HDACi with excellent pharmacokinetics and pharmacodynamic profiles are already “clinical-stage,” they could rapidly be employed in clinical trials.

## Figures and Tables

**Table 1:** Available therapies for EBV diseases.

Modalities	Classification	Comments
Drug treatment	Acyclovir/ganciclovir	Inhibits virus replication and induces cell killing. Effective only against lytic EBV infection.

Immunotherapy	Vaccination	Recombinant gp350-based vaccine, CTL epitope peptide-based vaccine.
	Treatment of disease	Monoclonal antibody against CD21, anti CD-20 antibody (Rituximab); EBV-specific cytotoxic T cells from donor or *ex vivo* amplification of CTLs from patients and infusion back to the patient.

Virus-directed approaches	Utilization of presence of virus in the tumor	Various cell-killing strategies that are dependent on *EBV* gene expression.

**Table 2:** Virus-directed novel approaches.

Classes	Comments	Reference
Targeting EBV episome	Low dose hydroxyurea treatment	Chodosh et al. [50], Slobod et al. [51]

Inhibition of EBV transforming protein	Antisense RNA against LMP-1 oncoprotein	Kenney et al. [52]
EBV-dependent expression of cellular toxins	Expression of detrimental cellular proteins through OriP dependent expression vector	Hirai et al. [53], Kenney et al. [54]

Combination therapy	Induction of EBV lytic replication + cytotoxic drugs	Numerous, Listed in Table 3

**Table 3:** Combination therapy approaches in the treatment of EBV malignancies.

Lytic replication inducer	Drug	Target cells	*In vivo*	Reference
DNA methylase transferase inhibitors				
5-Azacytidine	GCV and 5-bromodeoxyuridine	EBV+ and EBV- BL cells	None	Moore et al. [75]

HDAC inhibitors				
Arg-Butyrate	GCV	LCL from lung transplant recipient	Single human patient	Mentzer et al. [70]
Arg-Butyrate	GCV		10 human patients	Mentzer et al. [76]
Arg-Butyrate	GCV		15 human patients	Perrine et al. [74]
Valproic Acid*	Cisplatin, 5-FU, Gemcitabine, Doxorubicin	LCL, gastric carcinoma-EBV, NPC	SCID mice	Feng et al. [77]
Na-Butyrate	GCV	P3HR1	None	Ghosh et al. [78]

Radiation				
*γ*-Radiation + Na-butyrate	GCV & AZT	LCL and BL cell lines	SCID mice	Westphal et al. [71]
*γ*-Radiation	AZT+GCV	LCL-4A1A	Nude rats	Roychowdhury et al. [79]

B-cell receptor blockade				
Rituximab+Dexamethasone	GCV	AKATA	Nude mice	Daibata et al. [80]

Proteasome inhibitor				
Bortezomib	^131^I-FIAU	BL cell line	SCID xenograft	Fu et al. [81]

Other				
Cis-platinum, 5-fluorouracil, Taxol	GCV	Gastric carcinoma, NPC	Nude mice	Feng et al. [82]
Gemcitabine and Doxorubicin	GCV	LCL, and BL cell lines	SCID mice	Feng et al. [83]
